# The Inter-Relation of Corporate Social Responsibility at Employee Level, Servant Leadership, and Innovative Work Behavior in the Time of Crisis from the Healthcare Sector of Pakistan

**DOI:** 10.3390/ijerph18094608

**Published:** 2021-04-27

**Authors:** Naveed Ahmad, Miklas Scholz, Muhammad Zulqarnain Arshad, Syed Khuram Ali Jafri, Raja Irfan Sabir, Waris Ali Khan, Heesup Han

**Affiliations:** 1 Faculty of Management Studies, University of Central Punjab, Lahore 54000, Pakistan; irfan.sabir@ucp.edu.pk; 2 Department of Building and Environmental Technology, Division of Water Resources Engineering, Faculty of Engineering, Lund University, P.O. Box 118, 221 00 Lund, Sweden; 3 Department of Civil Engineering Science, Kingsway Campus, School of Civil Engineering and the Built Environment, University of Johannesburg, Aukland Park 2006, Johannesburg PO Box 524, South Africa; 4 Department of Town Planning, Engineering Networks and Systems, South Ural State University (National Research University), 76, Lenin prospekt, 454080 Chelyabinsk, Russia; 5 Department of Management Sciences, Lahore Garrison University, Lahore 54000, Pakistan; muhammad.zulqarnain11@gmail.com; 6 Department of Management Sciences, Bahria University, Lahore 54000, Pakistan; Skhurram.bulc@bahria.edu.pk; 7 Faculty of Business, Economics, and Accountancy, Universti Malaysia Sabah, Sabah 88400, Malaysia; warissalikhan@gmail.com; 8 College of Hospitality and Tourism Management, Sejong University, 98 Gunja-Dong, Gwanjin-Gu, Seoul 143-747, Korea; heesup.han@gmail.com

**Keywords:** servant leadership, CSR-E, innovative work behavior, healthcare sector, servant employee

## Abstract

Organizational crisis can serve as a base to provide an opportunity to an organization for enhancing individuals, organizations, and communities. The healthcare sector is one of those sectors that remains under continuous pressure to provide high-quality service delivery to the patients. Hence, the requirement of innovation for this sector is huge when compared to other sectors. The majority of the previous studies have investigated the phenomenon of CSR at the employee’s level (CSR-E) to influence employee behavior positively. However, the importance of CSR-E to enhance the innovative capability of the employees at the workplace is not well-explored in extant literature. Moreover, it is not clear from previous studies how the concept of servant leadership can explain the employee’s engagement towards innovative work behavior (EIB). Thus, the current survey aims to test the relationship of CSR-E and EIB in the healthcare sector of Pakistan with the mediating effect of servant leadership. The data of the current study were obtained through a self-administered (paper-pencil) survey and they were analyzed through the structural equation modeling (SEM) technique. The empirical results of SEM analysis revealed that CSR-E and EIB are positively related and servant leadership partially mediates this relationship. The findings of the current study will be helpful for policymakers to improve their understanding towards CSR-E to induce EIB in the time of crisis. At the same time, the current study also highlights the importance of servant leadership to the policymakers in encouraging the employees to display their innovative capability at the workplace to serve their organization during the time of crisis.

## 1. Introduction

Corporate social responsibility (CSR) has been of interest not only to the academician but also to business professionals [1]. CSR has become a reality in the industry and caring about the well-being of the environment, and the community has emerged as an important factor in organizational planning [2]. CSR is a strategic decision that an organization takes the environmental issues into account and is accountable to the community, for example, in the form of support and commitment to the local community [3]. Recent CSR approaches have changed the organization’s approach to revenue generation and improved their business profiles in terms of environmental, economic, social, legal, regulatory, and organizational objectives [4]. A plethora of previous studies have extensively explored CSR from a macro-level perspective [5,6,7,8]. However, the impact of CSR strategies at the level of employees (CSR-E) is not well-explored in previous literature. It is quite recent that research has shifted from the macro-level CSR to the micro-level, highlighting how CSR affects the attitudes and behaviors of employees [9,10,11]. Micro-CSR is a study of the results and experience of CSR strategies on employees at workplaces evaluated at different levels of individuals [12].

The dynamic business environment in the recent era has forced organizations to quickly adjust their strategies to become more competitive. Innovation is one of the most important strategies for organizations to ensure success [13]. It is worth mentioning here that employees are the real innovators, not the organizations, and perhaps this is the reason that recent research studies are stressing the importance of employees’ innovative work behavior (EIB) [14,15,16]. Successful organizations encourage their employees to display their innovative capabilities at workplaces [17]. EIB is a special form of employee behavior that is important for organizational outcomes including survival and growth [18]. An employee’s innovative work behavior is all about the initiation and implementation of an idea, and it is well-differentiated with the concept of creativity that only considers the novelty and usefulness of an idea [19].

Research has considerably established that CSR is helpful to achieve different employee-related outcomes, for example, employee’s satisfaction [20,21], employee’s psychological capital [22], employee’s well-being [23], employee’s commitment [24], employee’s engagement [25], etcetera. However, when compared to external stakeholders, studies on internal stakeholders (employees in the present case) are still sparse. In this context, few studies have been specifically conducted examining the effects of CSR on employee’s attitudes and behavior [26,27]. However, it is not clear from extant literature why CSR motivates employees to the desired behavior and attitudes. It is important to understand how CSR affects employee behavior and performance, as employees are directly involved in the implementation and execution of CSR measures and can consider resources that are related to CSR policy, depending on the impact and duration of this policy [28]. EIB is complex, because, when an employee introduces an idea, he or she has to face different hurdles, for example, insecurity, resistance from other employees, fear of failure, and lack of resources [29]. Therefore, it is even more important to look at the employee’s underlying mechanism for EIB.

**Figure 1:**
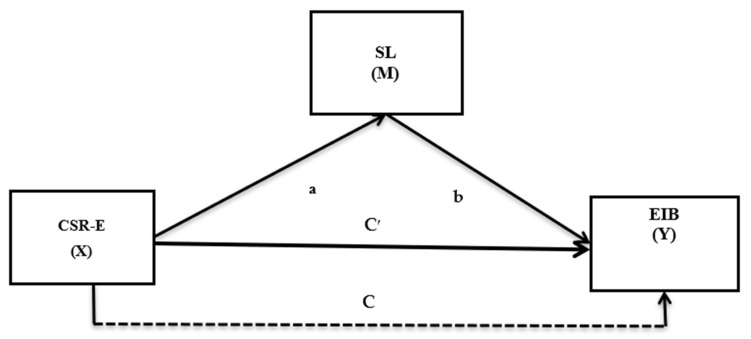
The proposed research model, based on the authors’ conception. This model comprises three variables, corporate social responsibility at the employee level (CSR-E) = the independent variable (X), servant leadership (SL) = the mediating variable (M), and employee innovative work behavior (EIB) = the dependent variable (Y).

CSR is an integral part of business strategies and it has a long-lasting impact on the employees to induce their performance [30]. CSR engagement of an organization promotes fairness and honesty at the workplace and employees believe that their organization will reliability and psychological security, where employees can act without fear of consequences and take risks [31]. Thus, CSR promotes a work environment that has a significant impact on employees’ innovative capability [32]. Moreover, different research studies have also established that the phenomenon of servant leadership promotes EIB [33,34,35]. However, it is not known from existing research studies how the concept of servant leadership and CSR-E can encourage the employees to display EIB. Therefore, exploring the relationship of CSR-E and EIB with a mediating effect of servant leadership in the healthcare sector of Pakistan during the time of crisis is the objective of the current survey. The proposed research model is presented in Figure 1.

The healthcare sector of Pakistan was purposefully selected to serve the objectives of the current survey due to some specific reasons. First, the healthcare sector of Pakistan is a kind of service industry that continuously faces a challenge to provide high-quality health-related services to patients. This high-quality service deliverance is only possible when this sector is willing to continuously adapt to new and innovative ways to serve the patients [36,37]. In this regard, the current research study argues that employees are an important source in providing innovative solutions in an organization and, hence, seeking to enhance EIB in healthcare organizations is not without logic. This importance of employees as a source of innovation is also supported by extant researchers [38,39,40]. Second, the healthcare sector represents a classic case in which the duration of employee–patient interaction is when as compared to other segments as healthcare employees keep in touch with the patients even for several weeks during their stay in a hospital [41,42]. Therefore, they can play an important role in innovation, because, during their interaction with patients, they observe and learn new things to perform a task innovatively. Likewise, they sometimes get new ideas from patients as well, which again highlights the importance of employees as a source of innovation for this sector. Third, the stiff situation of competitiveness in the healthcare sector of Pakistan also demands continuous innovation, because, through innovative ways of doing things, a hospital is capable of overrunning its rivals. Because, as per the findings of Porter [43], in an industry where the level of rivalry is high, innovation is a key strategic enabler that provides a strong competitive advantage to an organization over its rivals. 

There are some state-of-the-art contributions of the current survey to extant literature. First of all, the current survey adds to the existing literature of CSR and organizational management by acknowledging the employees as a source of innovation. The majority of the past studies have investigated the impact of CSR to achieve different organizational outcomes [44,45,46,47,48]; however, the relationship of CSR-E to foster EIB is barely addressed by extant researchers. Further, it is not clear from extant studies how servant leadership can be linked with EIB. Although there have been some studies on this topic [49,50,51], studies have produced mixed findings that highlight that there is more need for research in this area. Finally, the majority of the past studies have explored CSR relationships with employees in other sectors, like hospitality [52,53], banking sector [54], and small and medium-sized enterprises [55]; however, the healthcare sector did not receive due attention. The remainder of this article is composed in the following parts. The coming part discusses the literature review and theoretical support. After this, there comes the methodology part, in which the authors have discussed the sampling and data collection process along with instrument details. The last two parts discuss results and discussion for hypotheses testing, discussion, and implications.

## 2. Theory and Hypotheses

The current survey uses the lenses of social learning theory [56] and social identity theory [57] to explain the proposed relationship and formulate hypotheses. Social learning theory states that individuals learn different things by observing others. In this regard, when employees observe the helping behavior of their leader (servant leader) at the workplace, they imitate his behavior and practice this on their part. In the context of the current study, the employees are expected to help their organization by performing extra roles. One such extra role is to display the innovative capability of the employees. The servant leaders are likely to set an example of role-model for their employees by helping, promoting, and encouraging the employees at workplaces. Similarly, CSR philosophy also stresses the betterment of different stakeholders, and employees are important internal stakeholders. Thus, an organization that follows CSR principles, is expected to help and support its workers. In response, employees feel an extraordinary motivation to help their organization and, hence, they try their level best to think of new and innovative ways to help their organization. Moreover, social identity theory focuses on the interplay between personal and social identities. This theory attempts to explain and predict different circumstances for which people think of themselves as individuals or as a member of certain groups. In other words, social identity theory states that individuals are expected to categorize others based on some characteristics. In the scenario of the current survey, as the servant leader focuses on the betterment of followers, thus a strong bond of belongingness is developed that encourages motivating the employees to advance their thinking to achieve different organizational goals. Hence, the dyadic relationship between a servant leader and the employee works as a base of motivation for employees to display their innovative capabilities to enhance the overall efficiency of the group that they belong to or they identify themselves (the organization in this case).

Businesses that follow CSR principles focus on remaining profitable, comply with the law, adhere to the code of ethics, protect the environment, and ensure the larger benefit of society [58]. The current research study defines CSR as per the definition of Carroll [59], who described it as the actions taken by organizations to take care of diverse stakeholders, such as the environment, the community, and government agencies. Based on this definition, the organization must work to protect the well-being of the entire community and improve its interests and the preservation and care of nature [60]. Organizations need to publish CSR strategies and inform stakeholders about their genuine efforts to improve society and the environment [28]. It is worth mentioning that earning a positive CSR perception from stakeholders is very important for every organization. This is important because if stakeholders’ CSR perception of an organization is not positive, they will feel that the organization’s engagement in CSR is only symbolic, which leads to igniting negativity among stakeholders [61]. In this regard, employees are one of the most important stakeholders in an organization, so their CSR perspective is very important [62]. EIB is defined as “the behavior of individuals intended to initiate and deliberately create new and useful ideas or processes at the workplace” [63]. Employees’ CSR perceptions of an organization positively influence their behavior [26,64]. Employees are proud to be a member of an organization when they see that the organization is engaged in CSR for the benefit of society and the environment and is ethically responsible [65]. The CSR engagement of an organization creates a sense of confidence and security, where employees can act without fear of consequences and take risks [66]. When employees are risk-takers, they are not inclined to perform routine tasks, rather, they are engaged to invent new ways of doing things at workplaces, which ultimately enhances their innovative capability [17,67].

The atmosphere of the workplace has a significant impact on the innovative capability of employees [68]. In an organization where the safety and mental stability of employees are encouraged, it is likely to expect that employees to come up with new and innovative ideas. A plethora of previous research studies establishes that CSR-E is positively related to EIB [31,53,69,70]. The CSR activities of an organization foster an environment of confidence among the employees and this sense of confidence urges them to take risks and think about innovative ways to induce organizational effectiveness [71]. Employees who see that an organization’s CSR efforts are focused on improving the community and the environment have a greater sense of ‘meaningful work’, which, in turn, improves productivity and creativity [72]. Effective socially responsible organizations provide their employees with open and free employment opportunities to produce innovative solutions for the organization [73]. As a result, an employee’s perception of CSR for the organization improves his motivation to try new ideas, and that they can take a step forward in the implementation of these ideas due to a supportive organizational environment [74]. Different researchers in the extant literature have also acknowledged that CSR is positively related to EIB. As an instance, Li, et al. [69], conducted a research study to investigate the impact of CSRE on service innovation performance with EIB as a mediating variable and came out with the results that CSRE directly, and via EIB, enhances EIB in the service sector. Likewise, Li, Zhang, Wu, and Peng [70] verified, in their research study, that CSR-E can foster the innovative behavior of employees at the workplace in Taiwan. Moreover, Ratajczak and Szutowski [75] also confirmed that an organization’s CSR activities are directly related to employee’s innovation performance. Several other scholars also hold the same argument that CSR is positively related to EIB [76,77,78,79].

In line with social identity theory, when employees recognize the social responsibility of the organization in which they work, they positively identify themselves with such organizations and they are urged to enhance the overall progress of their organization by thinking of new ways to perform different organizational tasks. Given the above discussion, it is established that, when an employee realizes that the organization is involved in CSR for the betterment of society and the environment, it may affect work-related behavior such as EIB. Thus, the authors propose the following hypothesis.

**Hypothesis** **1** **(H1).** *CSR-E and EIB are positively related*.

Servant leadership is a style of leadership in which corporate leaders serve and help others to achieve different development opportunities, prepare subordinates for their best, and, ultimately, support their organization in achieving organizational success [80]. Servant leadership is a concept that is rooted in the philosophy of “serving others”. A servant leader puts the needs and interests of his followers first and is focused on caring for others, including society as a whole [81]. This description reflects the extent to which a servant leader has a deliberate focus on the interests and well-being, relationships with employees, organizational care, and the larger benefit of the community [82]. Serving others comes first in servant leadership. They deliver guidance to the followers for their development and growth and they have a positive impact on their behavior, ethics, and performance [83]. Previous research has confirmed that servant leadership has a positive impact on EIB [35,49,50,84], which, in turn, helps to understand the relationship between employee leadership and EIB. 

An important drive of servant leadership is to serve stakeholders and, hence, a servant leader sets an example to the followers to become a “servant employee” who tries to help the organization by engaging himself in innovative activities [50]. Innovation is multi-faceted in that it requires different tasks and individual behavior at each stage, involving the generation, promotion, and implementation of new ideas. During this process, a person first comes up with ideas or solutions to the identified problems. These ideas and solutions can be borrowed from different sources. The later step is to come up with a legitimate approach or solution by seeking the support of the organization [85]. Past literature in the field of organizational ethics has shown that the formation of a strong work ethic creates positive ethics and helping behavior among employees [86,87]. In particular, the innovative capability of employees is closely linked to the ethical context, as the values of “accepted work ethic” grow in the context of “participatory thinking” related to innovation, which is regarded as the ability of employees to evaluate, understanding of the situation, as well as the workplace as a whole [88,89]. 

By the same token, the concept of CSR also stresses caring for others (society, stakeholders, nature, etc.) and, hence, an organization’s engagement in CSR activities inculcates a sense of caring among workers [90]. Employees working for a socially responsible organization are likely to develop a kind of spiritual consciousness that ultimately encourages them towards creativity and innovation [73]. According to the theory of social learning, employees perceive their servant leaders as role models and imitate their helping behavior on their part. Thus, because of the leadership effect of a servant leader, the behavior of employees is also molded, and they want to engage themselves in extra-role behaviors (innovative work behavior) to help their organization to achieve business excellence. Moreover, as per social identity theory, the employees feel positive to identify themselves with a socially responsible organization. Thus, as a member of such an organization, they put every effort to enhance the group image (organizational image). Hence, CSR-E and servant leadership both support employees to be engaged in EIB. The above discussion and theoretical support lead the authors to frame the following hypotheses.

**Hypothesis** **2** **(H2).** *Servant leadership is positively related to EIB*.

**Hypothesis** **3** **(H3).** *Servant leadership mediates between CSR-E and EIB*.

## 3. Methodology

The current research study selected the healthcare sector of Pakistan to test the proposed relationships. To do this, the authors selected four large hospitals from the city of Lahore in Pakistan. These four hospitals included Hijaz hospital (HH), Pakistan Kidney and Liver Institute and Research Centre (PKLI), Shaukat Khanum Memorial Cancer Hospital and Research Centre (SKMCH&RC), and Iqraa Medical Complex. There were specific reasons for choosing these hospitals, such as all of these hospitals are engaged in different CSR-related activities. Moreover, these are state-of-the-art hospitals that deal with a large number of patients around the clock and employ thousands of employees. Likewise, these hospitals are actively engaged in arranging different training sessions for their employees for their skill development, which shows they are concerned with employees. Lastly, these hospitals use, up-to-date technology in their procedures that is an indication of their innovation preference. Hence, the selection of these hospitals is not without logic.

Before starting the actual data analysis phase, the authors contacted spokespersons of the selected hospitals to seek their support and permission to collect the data from their staff. The authors also signed an agreement with the ethical bodies of these hospitals to maintain ethical standards in the process of data collection. Further, the authors obtained informed consent from every respondent to participate in the survey voluntarily. The respondents were also given a choice to quit the survey at any stage if they do not feel comfortable. After seeking formal approval from the officials of each hospital, the authors arranged for the data collection process. The wake of Covid-19 posed serious challenges for the authors during the process of data collection and, hence, the authors had to arrange for special protocols in this regard. Thus, the authors had to stay for long hours in hospitals for the sake of data collection. The data were collected from the respondents during January 2021. The authors distributed a total of 900 surveys among the respondents of these four hospitals and received back 431 surveys from different respondents. Hence, the response rate of the current survey was 47.88%. The data were collected in two waves with a time-lagged difference of two weeks. This study followed the ethical guidelines given in Helsinki Declaration. The authors also obtained approval from the ethical committee of PKLI. 

### Measures and Handling of Social Desirability

The current survey used already established scales to measures the constructs. Thus, the issue of validity and reliability was non-existent here, because adapted scales have their pre-established validity and reliability. The scale of CSR-E was taken from Schaufeli and Bakker [91], which consisted of three items. Similarly, a seven-item scale of servant leadership was taken from the study of Liden, et al. [92]; likewise, the scale of EIB was taken from Hu, et al. [93], and this scale consisted of six items. The authors used a five-point Likert scale for the current survey. 

The authors took several measures to address the issue of social desirability. For example, the survey items were randomly scattered throughout the questionnaire. The authors did this in order to break any sequence of answering the responses by the respondents. This step is also helpful in dealing with the likelihood of any liking and disliking for a particular construct. Likewise, the instrument was checked for accuracy and suitability by experts in the field. This step is necessary in order to address any ambiguity or confusion in any item statement due to complex or dual-meaning words. Likewise, the authors requested the respondents for their true response, so that the findings generated by their input may reflect the reality. Different scholars also recommend these steps to mitigate the level of social desirability [10,11,94,95]. Table 1 presents the demographic detail of the sample.

## 4. Results

### 4.1. Common Method Variance

Since all of the information in the current survey was obtained from the same individual, there is a possibility that the issue of common method variance (CMV) may exist in the dataset. Hence, to validate whether the issue of CMV does exist, the authors performed Harman single-factor-analysis (SFA). The authors allowed all of the items to converge on a single-factor to detect CMV, according to the guidelines of Harman [96]. The general guideline here is that, if the results of SFA confirm the presence of any single-factor that is dominant and explains more than 50% of the total variance, then it is evident that CMV is a potential issue to be addressed by the researchers. In the current scenario, the results of SFA confirmed that there is no such factor that explains more than 50% of the total variance. The largest variance that was explained in the case of the current survey was 44.68% which is less than 50%. Hence, based on the results of SFA it is verified that there is no issue of CMV in the dataset of the current survey. 

### 4.2. Convergent Validity, Factor Loadings, and the Reliability Analyses

The authors started the data analysis phase by performing different tests after validating that there is no issue of CMV in the current survey. The results of these tests are reported in Table 2, which includes the results of convergent validity, reliability analysis, and factor loadings. The convergent validity was assessed on the basis of average-variance-extracted (AVE) values. To achieve this, the authors took the sum of squares of all loadings and dividing by the number of items. For example, the sum of square loadings for CSR-E was 1.81, which was divided by the number of factors (1.81/3) that resulted in 0.60 as AVE value for CSR-E. The general rule for convergent validity is that if the value of AVE for a variable is larger than 0.5 then it is established that the criterion for convergent validity for that variable is fulfilled. Likewise, the authors have also reported the factor loading for each variable in Table 2. To this end, all of the loadings were well above the minimum threshold of 0.40. Lastly, Table 2 also contains the results of reliability that were observed through Cronbach alpha (*α*) values and composite reliability values (C.R). The α values were obtained through SPSS software whereas, C.R values were calculated using AMOS software during confirmatory factor analysis (CFA). It is to be noted that both reliability values are important to calculate, but Cronbach alpha (*α*) is an average measure of inter-item consistency that is preferred during exploratory factor analysis. However, calculating and reporting composite reliability is a modern way to conduct reliability analysis and it is more preferred by contemporary researchers. Both types of reliabilities were significant (both α and C.R were > 0.70)

In the next stage of data analysis, the authors validated whether the data-based model fits the theoretical model or not. To assess this, the authors performed CFA in AMOS and checked the results of different model-fit-indices (*χ*^2^/*df* = 3.521, RMSEA = 0.059, NFI = 0.961, CFI = 0.928, IFI = 0.922, TLI = 0.957, and GFI = 0.927). The results of model-fit-indices are reported in Table 3 for the readers against their acceptability range. The results validated that there is a good fit between theory and the data. Thus, there is no issue of model-fit in the dataset of the current survey. Table 3 also presents the results of correlation analysis. According to these outcomes, all constructs are showing positive correlations. As a case, one can see that the correlation between CSR-E and SL is 0.27 ^**^, which is positive and significant, confirming that these variables are positively related to each other. Next, the authors verified whether the criterion of discriminant validity (DSV) is established in the case of the dataset of the current study. To do this, the authors calculated the square-root of AVE (SQAVE) for each construct and compared it with the values of correlation in comparison. To explain further, the SQAVE value for CSR-E is 0.78, which is above the correlation values (0.27 **, 0.32 **). These results provide enough support to accept that the items of a construct discriminate with the items of other construct and, thus, the criterion of discriminant validity is established. 

### 4.3. Hypotheses Testing

The authors continued with the data analysis to validate the hypotheses of the current survey. To do this, the authors used the structural equation modeling (SEM) technique in AMOS. SEM is an advanced level technique for data analysis to evaluate complex models, as is the case with the current research study. Further, SEM analysis is a co-variance-based analysis approach that is very popular among contemporary researchers [97,98,99], due to its advanced level features when compared to the conventional regression-based technique of data analysis. The authors in this connection performed SEM analysis in two stages. The first stage of SEM started with checking the direct effect analysis in which there was no intervention of any mediator in the model. Table 4 shows the results of the direct effect model. As per these results, the direct effect model produced significant results. These results confirmed that the first two Hypotheses (H1) and (H2) of the current survey are supported. These outcomes were declared on the basis of beta estimates and *p*-values (*β*1 = 0.31, *β*2 = 0.38, *p <* 0.05). The results further validated that the effect of SL on EIB is stronger as compared to the effect of CSR-E on EIB. Moreover, the model-fit-indices were also significant in this regard (*χ*^2^/*df* = 3.192, RMSEA = 0.051, NFI = 0.968, CFI = 0.936, IFI = 0.931, TLI = 0.960, and GFI = 0.931). 

The second stage of SEM analysis was carried out with the inclusion of SL as the mediating variable. To test the effect of mediation, the authors use the bootstrapping method in AMOS by using a large bootstrapping sample of 2000. The bootstrapping method is more sophisticated as compared to the previous mediation analysis that was suggested by Baron and Kenny [100], which is criticized by different scholars, including Hayes [101] and Zhao, et al. [102]. The output of bootstrapping approach confirmed the mediation effect of SL between CSR-E and EIB. It is to be noted that the beta estimate, which was earlier 0.31 during the direct effect model (CSR-E → EIB), is reduced (*β*3 *=* 0.037 **), which is an indication that SL partially mediates between CSR-E and EIB. The further detail of bootstrapping is presented in Table 5 for the readers

## 5. Discussion and Implications

The current survey was carried out to serve two major objectives. The first objective of the current survey was to test the relationship between CSR-E and EIB in the healthcare sector of Pakistan in times of crisis. To this end, the empirical results of the current survey validated that CSR engagement of a hospital at the level of the employee helps to enhance the innovative behavior of employees at the workplace. The respondents of the current survey were of the opinion that, when they realize that their hospital is concerned to work for the betterment of society and the environment, they positively identify themselves with such a hospital. Thus, CSR-E positively relates to EIB in the context of the healthcare sector of Pakistan. Different researchers in the existing literature also support this argument that CSR-E and EIB are positively associated with each other [31,32,53,69]. This relationship can also be explained in the light of social identity theory. As per this theory, the employees working in a socially responsible hospital positively identify themselves as a member of the group (the hospital in this case). Thus, they put every effort into enhancing the effectiveness of their hospital via their engagement in different innovative activities. Further, the employees of a socially responsible organization develop a sense of security and fairness at the workplace and, hence, they do not hesitate to take risks for the betterment of their organization. Therefore, they think about new ideas without the fear of failure to better serve their organization and come up with new ideas especially in the time of crisis. 

The second objective of the current study was to test the mediating effect of Servant leadership between CSR-E and EIB. The empirical results in this regard confirmed that servant leadership partially mediates the relationship between CSR-E and EIB. The respondents of the current survey established that the helping behavior of their leader (servant leader) encourages them to adopt such behavior on their part. Thus, a servant leader helps to transmit the sense of “servant employees” among the followers. A servant employee, like a servant leader, puts the organizational interest at the fore-front and wants to help his organization through extra-role behavior. One example of such extra-role behavior is the innovative work behavior of employees. The current study is not the first one to confirm this relationship, as different previous scholars have also confirmed that a servant leader inculcates innovative work behavior in employees [35,50,83,84]. The theory of social learning can also be related to further explain this finding. From the perspective of social learning theory, when employees see that their leader keeps the benefits and development of employees at the fore-front, they also learn this behavior on their part. Hence, after learning this behavior from their servant leader, they are motivated to perform extra-role for their organization and think of new ways to perform a task in a hospital. Likewise, the CSR orientation of a hospital also promotes a sense of caring for others among the employees. Therefore, both CSR-E and servant leadership encourage the workers to display innovative behavior in the workplace.

The current survey has some important theoretical implications to the existing literature on CSR and organizational management. In this regard, the first theoretical contribution of the current research study is that it enriches the existing literature to promote EIB through CSR-E. In this context, there have been some studies confirming that CSR activities at the employee level help an organization to positively influence the behavior of employees [26,27,86]. However, the relationship between CSR-E and EIB is sparse in the extant literature. Accordingly, this theoretical contribution adds significantly to the existing literature. The second theoretical contribution of the current research study is that it introduces the concept of servant leadership as a mediator between the relationship of CSR-E and EIB. The majority of the previous studies have established the relationship of CSR-E and EIB [53,69] and servant leadership with EIB separately [35,50]. However, this study investigates their impact on EIB in a single model. Finally, the current study adds to the scant literature of CSR in the field of the healthcare sector, whereas the previous study in the majority, explored CSR in different sectors rather than focusing on the healthcare sector. 

The practical implications of the current survey are also important for policymakers, especially from the healthcare sector. In this regard, the findings of the current research study are of utmost importance for the healthcare sector to consider their employees as a source of innovation. This implication has a special consideration for the healthcare sector which is under continuous pressure from patients to provide high-quality services. The authors’ argument here is that, without innovation, this high-quality deliverance of service is challenging. The current state of innovation in the majority of healthcare organizations in Pakistan focuses on enhancing innovation through the adaptation of new technologies. Although it is important to consider the latest technology for a hospital to overrun the competitors, policymakers are suggested to note that employees are an even better and low-cost source of innovation. Moreover, as the employees in healthcare organizations interact with patients very closely, so it is likely to expect that, during this interaction, they can develop new ways (innovative) to better serve the patients. Another important practical implication of the current study is that it highlights the importance of servant leadership to encourage the employees at workplaces to be engaged in innovative work behavior. To this end, policymakers are suggested to arrange for special sessions and training with their managers to let them realize the importance of servant leadership for employee’s innovative work behavior. Last but not least, the policymakers can benefit from the findings of the current study to upgrade their understanding of CSR to encourage the employees at workplaces to be engaged in innovative behaviors.

### Limitations and Potential Research Directions

Although the existing study offers adequate grounds to accept the proposed research model and relationships among variables, some limitations will need to be addressed by future researchers. The first limitation of this analysis is that it attempts to explain employees’ behavior through CSR-E and servant leadership. Although these variables are important to consider in explaining the behavior of employees, it is worth mentioning here that individual behavior is quite complex to understand. Hence, future researchers are suggested to consider other important variables in the proposed model of this study. For example, psychological contract and job autonomy may be important variables for future researchers to better explain employee innovative behavior. Likewise, this research study only considered hospitals that were located in Lahore city and, thus, the geographical concentration increases questions regarding the generalizability of this research. As a way to deal with this limitation, the prospective researchers are encouraged to consider a diverse sample of hospitals from different cities. Another limitation of this analysis is that it used cross-sectional data and, hence, forecasting causality based on cross-sectional data entails specific risks. Thus, future studies will need to consider the longitudinal data design.

## 6. Conclusions

The current study highlights the importance of CSR engagement of a hospital at the level of employees to foster workplace innovation. In this regard, the policymakers from the healthcare sector of Pakistan are encouraged to rethink CSR as a strategic enabler to foster the EIB of a hospital. Currently, in the majority of the healthcare institutions of Pakistan, CSR is kept limited to the extent of philanthropic orientation. This research argues that this is the time to shift from this thinking of philanthropic orientation of CSR to new areas, including workplace innovation. Moreover, the policymakers of the healthcare organizations are also encouraged to have a special focus on the style of leadership in their organizations, because, as per the empirical findings of the current survey, an appropriate leadership style, like servant leadership, is necessary to cultivate an environment in which employees are encouraged to display their innovative capability at the workplace. 

## Figures and Tables

**Table 1:** Demographic detail.

Demographic	Frequency	%
**Gender**		
Male	246	57.08
Female	185	42.92
**Age-group (Year)**		
18–25	56	12.99
26–30	109	25.29
31–35	126	29.23
36–40	77	17.86
Above 40	63	14.62
**Experience (Years)**		
1–4	66	15.31
5–7	117	27.15
8–10	128	29.70
Above 10	120	27.84
**Total**	**431**	**100**

**Table 2:** Item loadings, convergent validity, and reliability results.

Item	Loadings	Square	S.S	AVE	*α*	C.R
CSR-E1	0.74	0.55				
CSR-E2	0.78	0.61				
CSR-E3	0.81	0.66	1.81	0.60	0.73	0.75
SL-1	0.69	0.48				
SL-2	0.73	0.53				
SL-3	0.78	0.61				
SL-4	0.71	0.50				
SL-5	0.81	0.66				
SL-6	0.76	0.58				
SL-7	0.77	0.59	3.94	0.56	0.78	0.77
EIB-1	0.66	0.44				
EIB-2	0.74	0.82				
EIB-3	0.72	0.82				
EIB-4	0.75	0.74				
EIB-5	0.68	0.66				
EIB-6	0.79	0.74	4.22	0.70	0.86	0.84

Notes: Loadings = factor loadings, α = Cronbach alpha, C.R = composite reliability, square = square of item loading, S.S = sum of square.

**Table 3:** Correlation, discriminant validity, and model fit indices.

Construct	Mean	S.D	CSR-E	SL	EIB
**CSR-E**	4.21	0.72	**0.78**	0.27 **	0.32 **
**SL**	4.07	0.68		**0.75**	0.35 **
**EIB**	4.29	0.53			**0.84**
Model fit indices	Range	Obtained	Model fit indices	Range	Obtained
*χ*^2^/*df*	5.00	3.521	IFI	0.90	0.922
RMSEA	0.08	0.059	TLI	0.95	0.957
NFI	0.95	0.961	GFI	0.90	0.927
CFI	0.90	0.928			

Notes: S.D = standard deviation, ** = significant values of correlation, bold diagonal = discriminant validity results.

**Table 4:** The results for Hypotheses (H1) and (H2).

Path	Estimates	S.E	CR	*p*-Value	ULCI	LLCI	Decision
CSR-E → EIB	(*β*1) 0.31 **	0.046	6.74	***	0.210	0.639	Approved
SL → EIB	(*β*2) 0.38 **	0.051	7.45	***	0.315	0.521	Approved
Model fit indices	Criteria	Obtained	Model fit indices	Range	Obtained	*R* ^2^	
*χ*^2^/*df*	5.00	3.192	IFI	0.90	0.931		0.24 * (H1)
							0.28 * (H2)
RMSEA	0.08	0.051	TLI	0.95	0.960		
NFI	0.95	0.968	GFI	0.90	0.931		
CFI	0.90	0.936					

Notes: ULCI = upper-limit confidence interval, LLCI = lower-limit confidence interval, **, ***, * = significant values.

**Table 5:** Mediation and moderation results for Hypotheses (H3) and (H4.)

Path	Estimates	S.E	Z-Score	*p*-Value	ULCI	LLCI	Decision
CSR-E → ELS → EPB	(*β*3) 0.037 **	0.017	2.18	***	0.119	0.216	Approved
Model fit indices	Criteria	Obtained	Model fit indices	Range	Obtained	*R* ^2^	
*χ*^2^/*df*	5.00	1.86	IFI	0.90	0.940		0.32
RMSEA	0.08	0.033	TLI	0.95	0.968		
NFI	0.95	0.978	GFI	0.90	0.947		
CFI	0.90	0.952					

Notes: ULCI = upper-limit confidence interval, LLCI = lower-limit confidence interval, **, ***, = significant values, S.E = standard error.

## Data Availability

The data will be made available on request from the corresponding author.
